# Epidemiological Analyses Reveal a High Incidence of Breast Cancer in Young Women in Brazil

**DOI:** 10.1200/GO.20.00440

**Published:** 2021-01-12

**Authors:** Leonardo Fleury Orlandini, Maria Vitória do Nascimento Antonio, Claiver Renato Espreafico Jr, Priscila Longhin Bosquesi, Omero Benedito Poli-Neto, Jurandyr Moreira de Andrade, Francisco José Cândido dos Reis, Daniel Guimarães Tiezzi

**Affiliations:** ^1^ Ribeirão Preto School of Medicine, Department of Gynecology and Obstetrics, Gynecologic Oncology and Breast Disease Division, University of São Paulo, Ribeirão Preto, São Paulo, Brazil; ^2^ Advanced Research Center in Medicine, Union of the Colleges of the Great Lakes (UNILAGO), São José do Rio Preto, São Paulo, Brazil

## Abstract

**PURPOSE:**

Breast cancer screening is not recommended for young women (< 40 years old); therefore, those diagnosed are more likely to have advanced and metastatic disease, reducing treatment outcomes. This study aimed to investigate breast cancer epidemiology among young women in Brazil.

**METHODS:**

Data from three publicly available databases and a cohort from a university hospital in Brazil were analyzed in a retrospective study. Descriptive statistics was performed on disease prevalence and stage distribution across age groups. Incidence was estimated using age-standardized incidence ratio. The impact of age in disease-specific survival was also analyzed.

**RESULTS:**

Invasive breast cancer prevalence data by age group revealed that 4.4% and 20.6% of patients were < 35 and < 45 years old, respectively. In the United States, this prevalence was 1.85% and 11.5%, respectively (odds ratio [OR], 2.2; *P* < .0001). The percentage of regional and metastatic diseases were higher in São Paulo State (Fundação Oncocentro de São Paulo [FOSP]) compared with the United States (45% and 9.8% *v* 29% and 5.7%, respectively; *P* < .0001). In FOSP, regional and metastatic disease prevalence were higher among young patients (53.5% and 11.3%, respectively). The median tumor size in patients < 40 years old was higher (25.0 mm × 20.9 mm; *P* < .0001), and young patients have higher risk to be diagnosed with positive lymph nodes (OR, 1.5; *P* = .004) and higher proportion of luminal-B and triple-negative (TNBC) tumors. Young patients have a poor disease-specific survival because of late-stage diagnosis and more aggressive breast cancer subtypes (human epidermal growth factor receptor 2–enriched and TNBC) (*P* < .0001).

**CONCLUSION:**

In Brazil, breast cancer prevalence among young patients and late-stage incidence during this age span is higher. Advanced disease and more aggressive subtypes lead to a significant impact on breast cancer-specific survival in young patients.

## INTRODUCTION

Breast cancer is the leading malignancy affecting women worldwide.^1^ The probability of developing breast cancer increases with age, and the incidence of the disease is reported as uncommon in women younger than 40 years.^2^ According to the US SEER database staging system, the median age at diagnosis is 62 years, and the prevalence in patients < 35 years and between 35 and 45 years of age is 1.9% and 8.4%, respectively.^3^

**CONTEXT**

**Key Objective**
This study aimed to explore the incidence, characteristics at presentation, and outcomes of breast cancer in young women in Brazil. We performed epidemiological data analysis of three breast cancer databases from Brazil and have made comparisons with the US SEER database staging system.
**Knowledge Generated**
We observed a higher prevalence of breast cancer in young women (< 40 years old) in Brazil. Based on age-adjusted incidence, our results support the fact that the higher prevalence is because of a higher incidence. Young patients had more aggressive subtypes and higher risk of being diagnosed with advanced and metastatic tumors. Late diagnosis and the higher prevalence of triple-negative and human epidermal growth factor receptor 2 (HER2)–positive tumors in young patients are associated with worse disease-specific survival.
**Relevance**
Clinical-pathological characteristics of breast cancer in young women lead to significant impact on disease-specific survival. These findings add important information for public health planners and reinforce the need to identify specific surveillance and screening policies in young women.

Although stage has been described as the main prognostic factor in breast cancer, the disease is considered heterogeneous.^4^ Many studies have reported that molecular classification based on whole gene expression profile or immunohistochemistry could stratify patients into distinct subtypes in terms of biological behavior and prognosis.^5^ Moreover, young patients with breast cancer are more likely to be diagnosed with advanced disease, with the prevalence of aggressive tumor subtypes being higher compared with older patients.^6^ This scenario may influence the survival rate and the cost of treatment.^7^ Additionally, breast cancer in young patients has other critical implications; these patients are at high risk of developing a new breast cancer in the residual ipsilateral or the contralateral mammary gland because of the longer remaining lifespan and the social and economic impacts of the treatment for those at the working and reproductive ages.^8^

Breast cancer incidence is reportedly increasing in several low- and middle-income countries.^9,10^ This increase has been affecting all age groups; hence, determining the actual prevalence of breast cancer in women under screening ages is crucial for making public health policy decisions. In this study, we aimed to analyze the prevalence and incidence of breast cancer in young patients and its impact on disease-specific survival in a Brazilian population.

## METHODS

### Study Design

This was a retrospective observational study based on the analysis of three publicly available databases (Fundação Oncocentro de São Paulo [FOSP] cohort, the Instituto Nacional de Câncer [INCA] cohort, and the SEER Program–SEER cohort) and a cohort from Hospital das Clinicas–Ribeirão Preto School of Medicine–University of São Paulo, Brazil (HCRP cohort). It was approved by the local committee in ethics (#2.638.453/2018). The public databases were used to estimate breast cancer prevalence and incidence in Brazil and the United States. The HCRP database was used to analyze disease-specific survival in young patients with breast cancer. All patients included in the three Brazilian data sets had their treatment covered by the public health system (Sistema Único de Saúde).

### Population and Exclusion Criteria

A total of 114,936, 218,053, 1,632,850, and 1,970 patients were classified as International Classification of Diseases code 50 in FOSP, INCA, SEER, and HCRP cohorts, respectively. Data curation was based on histological diagnosis, year of diagnosis, sex, and registry duplication. The exclusion criteria were as follows: (1) duplication, (2) misclassification, (3) misdiagnosis, (4) diagnosis of noninvasive disease, (5) malignant tumor not arising from mammary epithelial cells, (6) diagnosis in male, and (7) year of diagnosis before the year 2000. Information without the year of diagnosis and years with small representation data (< 40% of the median) were also excluded. Table 1 presents the final cohort population.

**TABLE 1:**
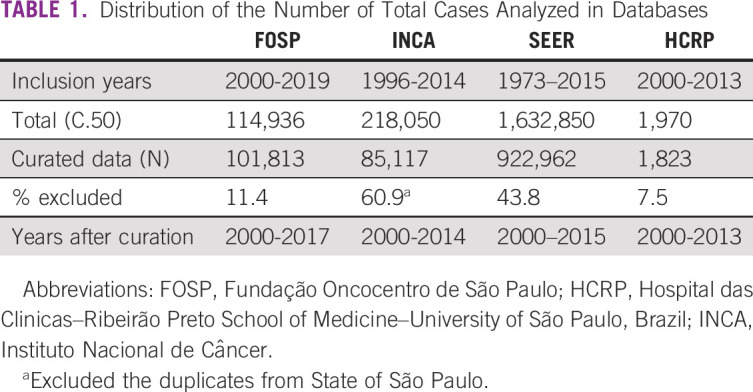
Distribution of the Number of Total Cases Analyzed in Databases

### Statistical Analyses

Patients were classified into age groups (5 years’ range). Descriptive statistics was performed. Disease prevalence and stage distribution across age groups were compared using χ^2^ test. We used 2010 population estimates in São Paulo, Brazil, and in the United States as the base for population incidence estimation.^11^ The age-standardized incidence ratio and the cumulative risk were estimated as previously described.^12^ To maintain stage classification uniformity, we used the SEER nomenclature: localized (stage I and II N0), regional (stages II with N1 and III), and distant (stage IV).^13^ For survival analysis, patients were divided into two groups as defined in the European Society for Medical Oncology 3^rd^ international consensus: young (< 40 years) and standard (≥ 40 years).^14^ The breast cancer subtypes in the HCRP data set were considered to be (1) luminal A if estrogen receptor (ER)–positive and/or progesterone receptor (PR)–positive, human epidermal growth factor receptor 2 (HER2)–negative, and grade ≠ 3; (2) luminal B if ER–positive while PR– and HER2–negative or if ER– and PR–positive, HER2–negative, and grade 3; (3) luminal/HER2 if ER–positive and/or PR–positive while HER2–positive; (4) HER2 if ER– and PR–negative while HER2–positive; and (5) triple-negative (TNBC) if ER–, PR–, and HER2–negative.^15^ The difference in primary tumor size was analyzed using Wilcoxon test. Chi-squared or Fisher's exact tests were performed to analyze association among qualitative variables and the estimated odds ratio (OR). Univariate survival analyses were based on Kaplan-Meier curves and tested for significance using the logrank test. Cox multivariate regression model including the significant variables from the univariate analysis was used for hazard ratios estimation. The level of significance was set at 5%. We conducted all analyses using R version 3.6.1.^16^

## RESULTS

### Prevalence of Invasive Breast Cancer According to Age (FOSP Plus INCA Databases)

**FIG 1:**
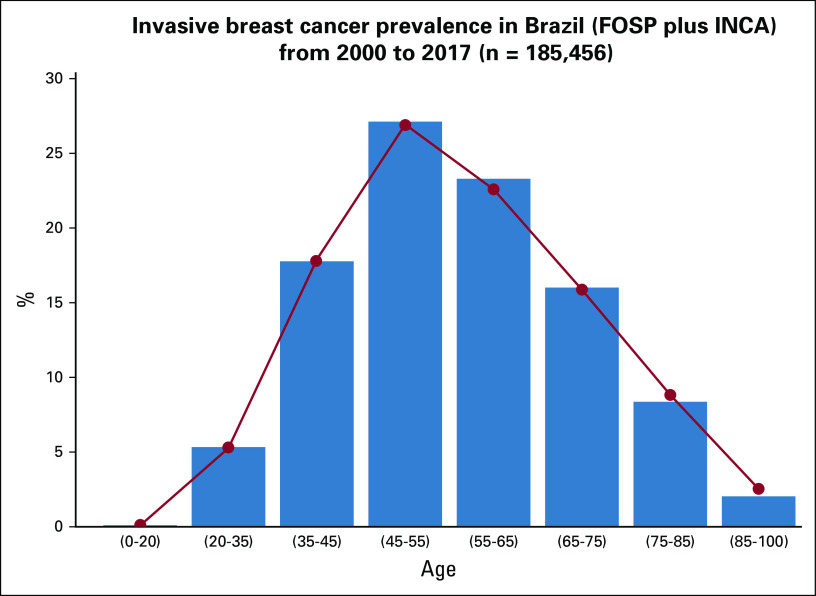
Breast cancer prevalence according to age groups in Brazil. FOSP, Fundação Oncocentro de São Paulo; INCA, Instituto Nacional de Câncer.

According to data from the FOSP and INCA databases, the median number of invasive breast cancer diagnosed per year from 2000 to 2017 was 5,183.5 (interquartile range [IQR], 2,021) and 5,841 (IQR, 1,002), respectively. The mean age at diagnosis was 52.3 ± 13.6 years (FOSP cohort) and 56.7 ± 14.3 years (INCA cohort). According to the prevalence of invasive breast cancer by age groups, in the FOSP cohort, 4.4% of patients were < 35 years and 20.5% were < 45 years. The INCA cohort showed a similar distribution, with 4.4% of patients < 35 years and 20.6% < 45 years. Figure 1 illustrates the distribution of invasive breast cancer across age groups in the FOSP and INCA databases.

### Comparative Analysis Between Brazilian Databases (FOSP Plus INCA) and the US Database (SEER)

According to the SEER data, the median number of cases per year was 57,202 (IQR, 7,142.25), and the mean age at diagnosis was 61.7 ± 14.0 years. The age at diagnosis is significantly higher in the SEER data set compared with the Brazilian data (*P* < .0001). The prevalence of patients with invasive breast cancer was 1.85% and 11.5% for women < 35 and 45 years, respectively. Breast cancer among young women accounts for 10.5% and 5% of cases in the Brazilian and SEER cohorts, respectively. There is a significant difference in prevalence among young patients between the two data sets (OR, 2.2; 95% CI, 2.20 to 2.28; *P* < .0001).

**FIG 2:**
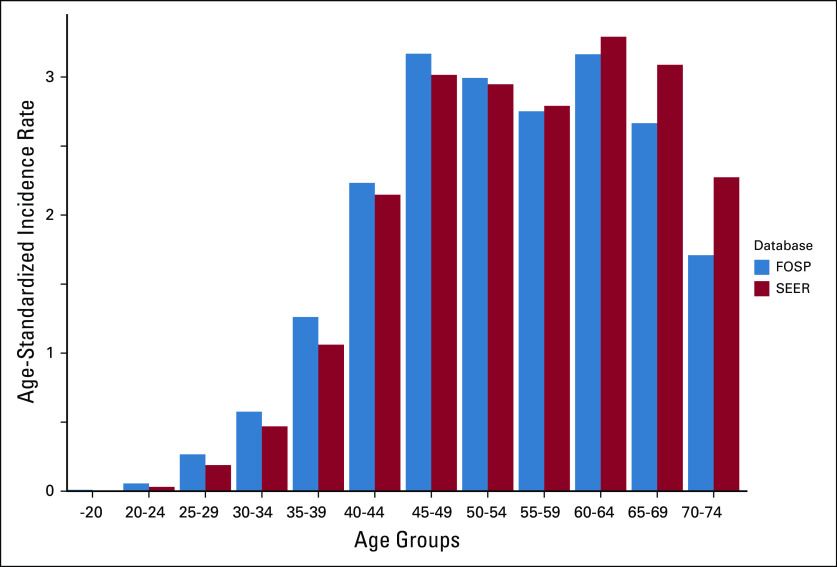
Age-standardized breast cancer incidence in Brazil (FOSP) and the United States (SEER). FOSP, Fundação Oncocentro de São Paulo.

To correct for population density in the distribution data, we used the SEER and FOSP data and estimated the age-standardized incidence ratios. The overall incidence ratio was higher in the SEER cohort (36.7 *v* 26.7 cases/100,000 women/year), with a cumulative risk by the age of 40 years of 0.13% (SEER cohort) and 0.15% (FOSP cohort). Figure 2 illustrates the age-standardized incidence ratios in both databases. Note that the incidence ratio by the age of 50 years is higher in the FOSP cohort.

### Stage Distribution Among Young Patients

**FIG 3:**
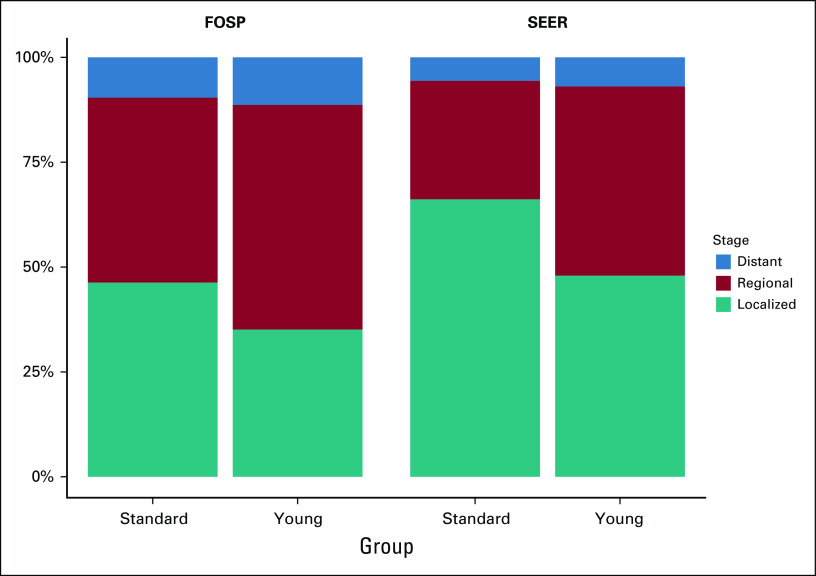
Percentage of localized, regional (node positive) and distant (metastatic) breast cancer patients in Brazil (FOSP) and the United States (SEER) according to age status. Standard ≥ 40 years old and young < 40 years old. FOSP, Fundação Oncocentro de São Paulo.

We analyzed the distribution of tumor stages at diagnosis according to the age at breast cancer onset using data from the SEER and FOSP databases. Overall, the percentages of regional and metastatic diseases are higher in FOSP cohort (45% and 9.8% *v* 29% and 5.7%, respectively; *P* < .0001). The prevalence of regional and metastatic diseases is even higher among young patients. In the FOSP cohort, 53.5% and 11.3% of young patients were diagnosed with regional disease and metastatic disease, respectively. Figure 3 illustrates the distribution of stages according to age groups in both databases.

### Impact of Age in Breast Cancer Subtypes and Survival

**FIG 4:**
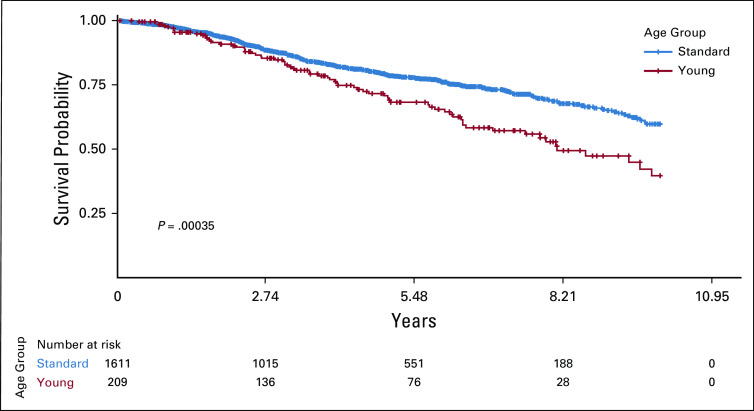
Disease specific survival according to age groups (standard versus young) in 1,820 breast cancer patients treated at Hospital das Clínicas, Ribeirão Preto School of Medicine, University of São Paulo, Brazil (HCRP cohort). Standard ≥ 40 years old and young < 40 years old.

We analyzed the association of age groups, breast cancer subtypes, and the impact in overall and disease-specific survivals using data from the HCRP database. Although the overall survival was not affected by age (*P* = .6), young patients have a significant reduction in disease-specific survival. The median survival time was 8.1 years (95% CI, 6.9 to 11.0) for young and 11.5 years (95% CI, 10.6 to 13.3) for standard patients (*P* = .004). Figure 4 shows the Kaplan-Meier curves for disease-specific survival.

Based on the univariate analyses, disease stage and tumor subtype significantly influenced the disease-specific survival. The distribution of breast cancer subtypes and stages are significantly different in young patients. Table 2 summarizes patient characteristics according to age group.

**TABLE 2:**
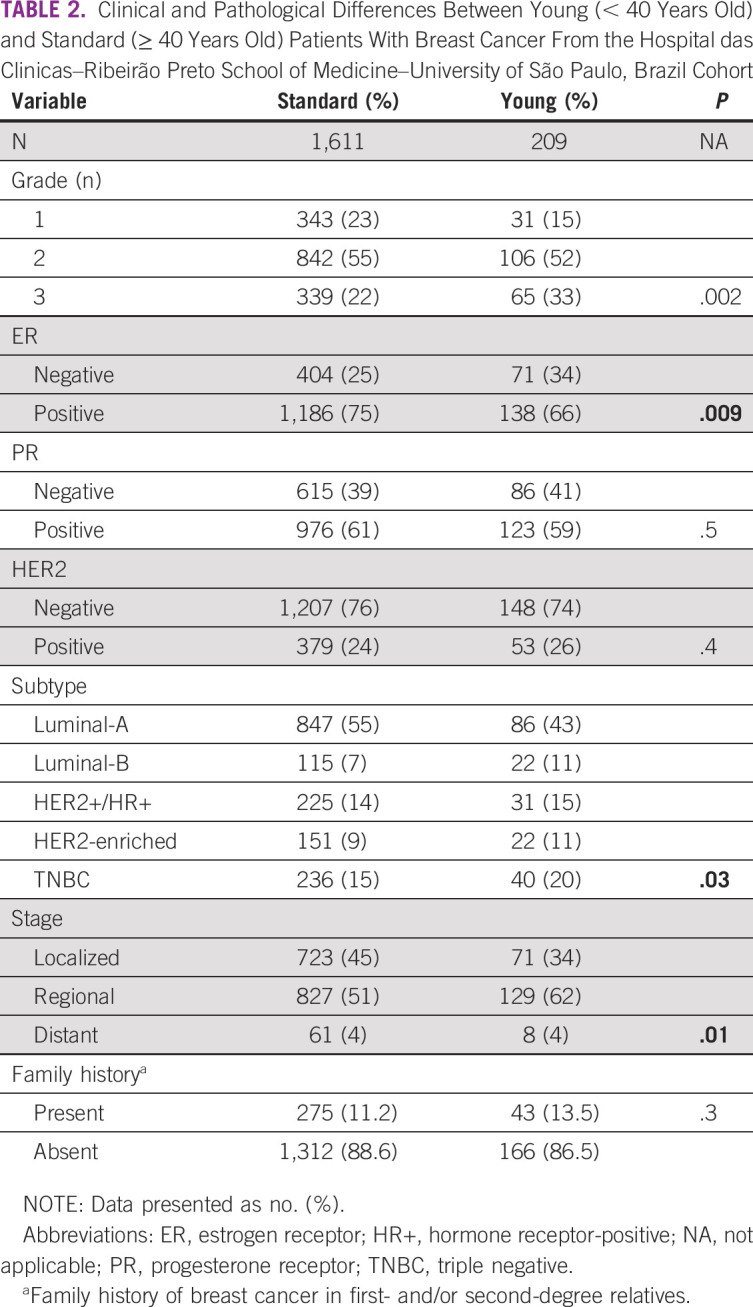
Clinical and Pathological Differences Between Young (< 40 Years Old) and Standard (≥ 40 Years Old) Patients With Breast Cancer From the Hospital das Clinicas–Ribeirão Preto School of Medicine–University of São Paulo, Brazil Cohort

Young patients presented with more advanced disease. The median tumor size in young and standard groups was 25.0 mm (IQR, 25) and 20.9 mm (IQR, 16), respectively (*P* < .0001). The axillary involvement was associated to the primary tumor size (*P* < .0001), and young patients had higher risk to be diagnosed with positive lymph nodes (OR, 1.5; 95% CI, 1.1 to 2.1; *P* = .004). Additionally, there was a higher proportion of luminal-B and TNBC among young versus standard patients (11% *v* 7.3% and 20% *v* 15%, respectively; *P* = .03).

To correct for age, stage, and tumor subtypes in the hazard ratios, we used the Cox proportional hazard ratio model to assess the effect of these multiple variables on disease-specific survival. According to the multivariate analysis, the significant variables affecting disease-specific survival were tumor subtype (HER2-enriched and TNBC) and tumor stage (Table 3).

**TABLE 3:**
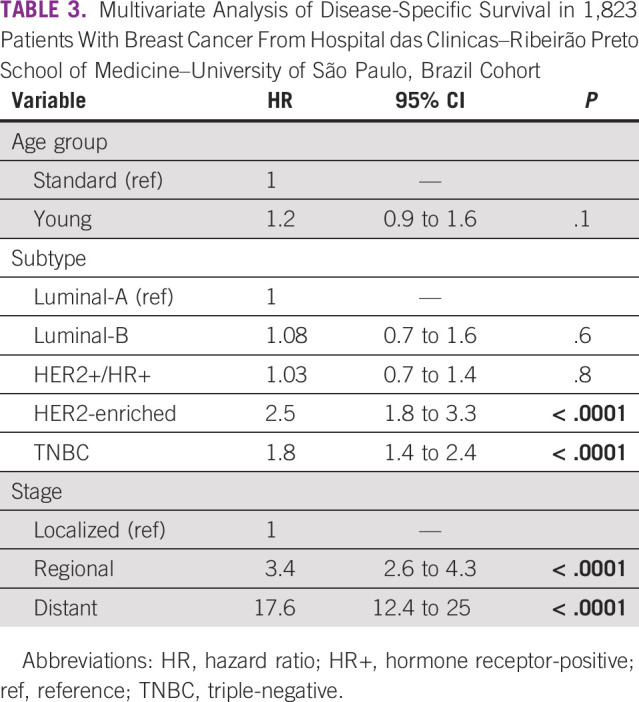
Multivariate Analysis of Disease-Specific Survival in 1,823 Patients With Breast Cancer From Hospital das Clinicas–Ribeirão Preto School of Medicine–University of São Paulo, Brazil Cohort

## DISCUSSION

Breast cancer diagnosis and treatment in young patients is challenging because aggressive subtypes are more frequent and most patients are diagnosed with advanced disease.^17,18^ In turn, this leads to a high cost of treatment, massive impact on familial, sexual, social, and economic aspects, and reduced survival rate.^8,19^ Although several data support the observation of a low breast cancer prevalence among young patients, we have demonstrated that the prevalence among the Brazilian population is not irrelevant. We identified 10.5% of diagnosed breast cancer in patients < 40 years. Additionally, the estimated age-standardized incidence suggested that our population is under higher risk of developing breast cancer before age 50 years. More than 60% of young patients were diagnosed with locally advanced or metastatic disease, and the prevalence of TNBC was also higher. These factors led to a significant decrease in disease-specific survival.

Breast cancer screening programs do not cover young women under average risk. There is no evidence that screening young women leads to a significant reduction in breast cancer mortality,^20,21^ added to the potential increase in overdiagnosis and the radiation-induced cancer.^21^ Furthermore, the screening mammogram's low sensitivity in dense breasts, in addition to the low disease prevalence, leads to an unacceptable predictive positive value in young women.^22^ Thus, in this age group, breast cancer is mainly diagnosed on symptomatic women.

Some studies have estimated the natural progression of breast cancer.^23,24^ Plevritis et al^25^ reported that the median tumor size during transition from local to regional disease (axillary involvement) is 2.5 cm. Among the HCRP cohort in our study, 50% of young patients were diagnosed with tumor > 2.5 cm, corroborating with the observation that most young Brazilian patients are diagnosed with regional and distant disease. Moreover, a previous study has shown that the difficulty in patient flow for public health care and the consequent timing between diagnosis and treatment are important factors associated with advanced clinical stage at diagnosis.^26^ Socioeconomic conditions, formal education, and race contribute considerably to this scenario, indicating that breast cancer awareness and access to the public health system are the main aspects that the government has to deal with.^27^ These observations are highly relevant for decision makers on public health policies.

In addition to the investment in cancer awareness and healthcare access, identifying people with high risk of developing breast cancer may be another strategy to optimize breast cancer care in young women.^21,28^ This approach aims to improve early diagnostic rates, based on increased surveillance and awareness, and to apply risk reduction strategies for high risk women.^29^ A cross-sectional study, using three breast cancer risk assessment tools (Gail, Tyrer-Cuzick, and BRCAPRO) and including 382 women 35-69 years of age in the South region of Brazil, has demonstrated that the tools can identify up to 8.8% of women 35-39 years as having high risk for breast cancer.^30^ These tools can also identify individuals at risk for Hereditary Breast and Ovary Cancer (HBOC) syndrome. One study has demonstrated the prevalence of *BRCA1* or *BRCA2* mutation in 21.5% of 349 unrelated individuals in Brazil with personal or familial high risk for HBOC syndrome.^31^ Integrating genetic cancer risk assessment in primary care with a genetic counselor is a potential opportunity for systematically screening high-risk families and individuals,^32,33^ which seems to be a rational measure for populations with high breast cancer incidence and prevalence.

Another point we would like to address is the higher incidence of breast cancer among young women in Brazil than in the United States, which is also supported by reports from Brazil and from other developing countries.^27,34,35^ Differences in disease incidence among young individuals may be attributable not only to inherited genetic factors but also to modifiable risk factors, such as smoking and alcohol, and the exposure to carcinogens. Although the etiological factors responsible for such differences are unknown,^17,27^ the chronic exposure to endocrine disrupting and carcinogenic pesticides may be a concern. Recent studies suggest the exposure of organochlorine pesticides may be associated with a higher incidence of breast cancer.^36^ Furthermore, toxicoproteomics analysis has demonstrated that women exposed to pesticides are more prone to develop ER-negative breast cancer.^37^ In terms of absolute numbers, Brazil is the largest consumer of pesticides worldwide.^38^ Therefore, future studies on population genomics and exposure to environmental carcinogens are necessary to elucidate these factors.

A recent study of the Brazilian population has already demonstrated unfavorable clinicopathological features for breast cancer among young women.^27^ However, this study did not analyze how these factors may influence the overall and disease-specific survival. In our study, we were able to explore the impact of molecular subtyping and stage in a long-term follow-up breast cancer cohort. Our data demonstrated that disease-specific survival is clearly affected by late diagnosis as well as TNBC and HER2-enriched breast cancer prevalence among the young population. The diagnosis of regional disease in young women increases the risk of dying because of breast cancer by 3.5 times. Such an observation reinforces the need to establish a program for the identification and surveillance of high-risk populations.

Our study has some limitations. First is the inherent limitation of retrospective studies, as well as the possibility of some missing data. Second is the lack of follow-up data among the FOSP, INCA, and SEER databases. Nevertheless, the follow-up information on the breast cancer patients under the HCRP cohort (n = 1,823) is highly consistent. Additionally, the age and stage distributions of the HCRP data set are highly similar to that of the FOSP and INCA databases, making this cohort a reliable representation of the Brazilian population.

In conclusion, we have demonstrated that the incidence of breast cancer in young women is higher in Brazil. The prevalence of patients with breast cancer in women younger the screening age is also high. Young patients with breast cancer are more likely to be diagnosed at the regional and distant stages, with a higher prevalence of more aggressive breast cancer subtypes, hence leading to a significant impact on disease-specific survival. Strategies to identify high-risk individuals in our population may be a starting point to drive specific surveillance and screening policies for breast cancer care and awareness for young women.
